# A Model to Promote Public Health by Adding Evidence-Based, Empathy-Enhancing Programs to All Undergraduate Health-care Curricula

**DOI:** 10.3389/fpubh.2017.00339

**Published:** 2017-12-11

**Authors:** Lon J. Van Winkle, Brian D. Schwartz, Nicole Michels

**Affiliations:** ^1^Rocky Vista University, Parker, CO, United States

**Keywords:** critical reflection, empathy, evidence-based medicine, health sciences curricula, relationship-centered care, service-learning, public health

## Abstract

Fostering empathy in future health-care providers through service-learning is emerging as central to public health promotion. Patients fare better when their caregivers have higher relationship-centered characteristics such as the ones measured by the Jefferson Scale of Empathy. Unfortunately, these characteristics often deteriorate during health-care professional training. Nevertheless, growing literature documents how we can promote empathy, and other patient-centered characteristics, throughout health-care professional students’ undergraduate education. As for proven treatment plans, we believe we should also use evidence-based guidelines to foster relationship-centered characteristics in our students when training them to practice as part of an interdisciplinary health-care team.

## Introduction

Empathy fosters public health in numerous ways. For example, higher empathy is associated with greater support for vaccination and other health precautions (1). In the case of child abuse, increasing empathy may promote public health at multiple levels (2). At the individual level, greater empathy might help potential offenders resist the urge to abuse children, and empathy could help parents respond better to children’s reports of abuse. Heightened empathy in health-care professionals may help them report suspicions of abuse instead of suppressing them. At the institutional and societal levels, higher empathy should cause leaders to advocate better for children rather than suppress reports of abuse and protect abusers (2). In regard to public health in general, greater empathy in health-care professionals improves patient outcomes and satisfaction regardless of the circumstances (3). In our opinion, we should use available evidence to foster empathy in health-care professional students as we do for other aspects of their training.

Health-care professionals use evidence to develop useful treatment guidelines for patients and to determine whether patients adhere to those recommendations. But how should we apply those guidelines to individual patients, and how do we help patients adhere? We believe health-care professionals need to gain patients’ trust in shared medical decision making. Trusting relationships with patients and health-care team members promote patient satisfaction, foster adherence with treatment plans, and minimize malpractice claims (4, 5). In our opinion, such trust requires empathy and compassion for each patient’s unique life experiences. When primary care physicians have higher Jefferson Scale of Empathy scores, their diabetic patients are 41% less likely to have acute metabolic complications and have an 80% greater chance of having good control of their blood hemoglobin A1c and LDL-cholesterol levels (6, 7).

Unfortunately, most health-care professional training programs lead to decreases or no changes in empathy rather than increases (3, 8). Nevertheless, methods to enhance empathy in health-care professional students are emerging (3, 9), and we believe such efforts should be our priority. Here, we consider methods used in selected studies for increasing empathy and related, relationship-centered, characteristics, and we present an evidence-based model for incorporation of these approaches into health sciences education programs throughout training.

## Studies Selected for Model Construction

### Students Entering Clinical Training Are Vulnerable to Loss of Empathy

Jefferson Scale of Empathy scores decrease dramatically in many medical students during their first year of clinical training in the United States (third year of undergraduate medical education) (3, 8, 10). This loss of cognitive empathy approaches crucial practical importance. One study (10) found an effect size (ES, *r* value) of nearly 0.5 for the loss of empathy in male medical students during the first year of clinical training (11, 12). In our opinion, interventions to increase or maintain empathy are best designed to span the entire period of undergraduate health-care professional education. As found in studies described in the next two subsections, relationships formed among students during their first year(s) of training can be used to help them support each other during the difficult first year of clinical education.

### The Need for Continuous, Long-term Interventions: Reinterpretation of the Results of Such an Approach

After 5 years of continuous training and support to enhance relationship-centeredness in undergraduate medical students in Belgium, Bombeke and associates (13) reported a decline in patient-centered attitudes among these students during the first year of their clinical training (i.e., their year-six clerkships). These investigators used six scales, including the Jefferson Scale of Empathy, to measure the patient/relationship-centered attitudes of their students. They attribute the students’ decline in patient-centered attitudes primarily to a “gap between ‘ideal’ training…and ‘real’ world” and to “problematic validity of measures” used in the study. For example, in the ideal world, students learned communication skills to use with patients, but the students observed a dearth of patient-centered communication in the year-six real world. Similarly, students claimed that “patients do not always want you to empathize” and that “the questionnaires (measuring empathy) do not capture that (patient preference).” While we might agree with the former statement especially in light of the hidden curriculum (14), students seem to be missing the meaning of survey questions regarding empathy in the latter case. In our opinion, to comprehend that some patients do not want empathy, students must first empathize with patients and learn their preferences. The Jefferson Scale of Empathy is designed to measure such dimensions of cognitive empathy. It may not be an oversimplification to conclude that medical students have an investment in their competence as caregivers and, like the rest of us, may deny events that call their caregiving competence into question. From our perspective of this study (and consideration of the studies cited below) the loss of patient-centered attitudes by students during their clinical clerkship might be due to a lack of continued empathy training and support to enhance relationship-centered beliefs during this sixth year of their medical education.

While the authors saw no difference between medical students trained and supported for five years to enhance patient-centered attitudes and those not trained, this intervention was actually quite successful when one views the authors’ data as a whole. Six measures of attitudes toward relationship-centered care were administered to students a week before the start of their clinical clerkships and at the end of the clerkships. When the pre-clerkship differences in scores by students receiving versus not receiving the intervention are compared, students trained to enhance their patient-centered attitudes scored higher on average on each of the six scales. When the six proportions, by which the trained group scored higher, are used in a paired *t*-test, the resultant comparison is statistically significant (*p* < 0.004). Moreover, the ES (*r* = 0.91) is of crucial practical importance (*r* > 0.50) (11). Unfortunately, this positive effect of patient-centered training was lost in the intervention group when the intervention and its support of students ceased during the sixth-year clinical clerkship (13). When such support of students is continued during the first year of clinical training, positive relationship-centered attitudes can be reinforced or even increased (15).

### Evidence That Interventions to Provide Peer Support Prevent Loss of (or Even Enhance) Empathy through Self-reflection by Students Entering Clinical Training

Principle clinical experiences at one clinical training site, with the same medical students, other health-care professionals, and patients reinforce patient-centered characteristics in medical students in the first year of clinical education (third year of medical school) in the United States (16). However, most medical schools do not have the resources to offer all students such training. In our opinion, many medical students likely feel isolated and confused when completing traditional, site-to-site and discipline-to-discipline rotations alone and in the context of the hidden curriculum during their first year of clinical education. Hence, health-care professions educators should work to provide support to students and foster professional development especially during this first year of frequent clinical experiences.

Important ways to reinforce professional development include self-reflection to foster empathy (17). For these reasons, Duke and associates (15) measured students’ growth in reflective ability and empathy during their first year of clinical training (third year of medical school) at Drexel University College of Medicine. They measured these aspects of growth before and after introducing a professional formation course into the curriculum of third-year medical students.

Professional formation had been a priority at Drexel in the first and second years of medical school, when small-group meetings were possible on campus. Upon introduction of the new course for third-year students, the same professional formation groups, which had met during the first and second years, could continue to meet in the third year using virtual technology. Thus, continuation of the support from their peers, which students had come to rely on during the first-half of their medical education, now continued through the difficult first year of clinical training.

In association, students’ scores on the Groningen Reflection Ability Scale increased dramatically over the third year of medical education (*p* < 0.0001), and in a manner that was of near crucial practical importance (ES, *r* = 0.445 for paired scores before and after the intervention). This scale measured three dimensions of reflection: self-reflection, empathic reflection, and reflective communication (18). Less dramatically, Jefferson Scale of Empathy scores also increased (*p* = 0.035 for a one-tailed *t*-test of paired values) as expected for such an intervention (3, 9). These results indicate that empathy, and other scores need not decrease with the onset of traditional clinical training.

### Use of Community Service As the Focus for Long-term Interventions

While various activities for reflection were used in the interventions to promote the professional development described earlier (13, 15), course- or curriculum-based community service promotes such development particularly well in our opinion. For example, medical students who performed community service (median of 4.6 h per year of medical school) had higher medical school grade point averages, USMLE Step 2 scores, and scores on residency director assessments (19). More importantly, medical students who performed community service had significantly higher Jefferson Scale of Empathy scores at graduation than their classmates who performed no service during their training (*p* < 0.0001) (20).

More broadly, course-based community service, including service learning, has consistently been shown to raise academic achievement as well as social and citizenship outcomes in K–12 and nonmedical higher education students (21–24). Introduction of critical thinking and reflection on community service by small teams of students in our medical biochemistry courses raised their empathy, patient-centered orientation, and examination scores. While reflection on other challenges of medical education and life fosters development of professional attitudes and behaviors, reflection on service learning by teams of medical students appears uniquely suited to promote empathy (25). In our opinion, continuation of such work by all health-care professional student teams throughout their undergraduate training should foster the highest levels of relationship-centered characteristics in these students. In this way, service-learning promotes public health (26, 27) in part because it fosters empathy.

## A Model Program for Intervention Throughout Undergraduate Health-Care Professional Education

### Program Description

To develop a program that promotes empathy and professional development among health-care professional students, we selected activities that, as reported in the literature, achieve these goals (discussed earlier). We propose development of a continuous team support system, reliant on reflection and virtual technology, centered on service learning, and starting at the beginning of undergraduate training. Although teams need not be interprofessional to be successful (13, 15, 25), we suggest forming teams of seven students from as many health-care professional disciplines as possible. For example, many institutions may be able to form teams comprised of medical, nursing, pharmacy, PT/OT, and physician assistant students. We believe the interprofessional nature of these support groups fosters development of core competencies for interprofessional collaborative practice (28).

The makeup of the teams depends on the institution and community partners. In our opinion and experience, teams can function well without facilitators (25, 28). Team meetings should commence shortly after matriculation of all groups of health-care professional students. These initial meetings allow team members to meet each other and organize their service-learning projects. Teams should select a team name and begin to consider possible projects. Students on a team can perform the same or different service learning. We believe requirements should include the following: (1) a minimum of 4 h of service learning by each student during an academic term and (2) after completion of service learning, student engagement in written critical reflection and consideration of how the learning relates to the content of courses within their program.

In our opinion, written critical reflection moves students past surface-level learning and supports professional development. These written reflections serve as a discussion point for team meetings as students reflect on significant and specific events. Significant events may include experiences that are challenging in some way or illustrate personal limitations, but events may also inspire, affirm, or awe (15). The critical reflection should create new meaning and understanding for each student.

The team setting provides peer support and a stage for discussion of each critical reflection. Teams should meet face-to-face for 90 min on four occasions each academic term. By organizing the times and venues of their own meetings, teams of seven students are able to discuss the written reflections of two members at each meeting and still have time to summarize all reflections at the final meeting of the term. In written reflections, students should consider how service learning enhanced understanding of professional expertise and responsibilities. This new understanding can extend to the roles and responsibilities of other health-care professionals when team membership includes students from more than one discipline. For example, for service learning performed at a nursing home, a PT student might explain the role of their profession in the management of a resident who is having knee replacement surgery. Teams should also write minutes of their meetings and members’ responses to the reflections of each other. Learning to set their own objectives and reflect as teams, as early in training as possible, seems essential in our opinion. Focus on individual reflection only in clinical settings may result in gaps in patient care due to inadequate reflection on team care (29). As students enter into clinical rotations, discussions can continue to take place via virtual classroom technology.

To maximize the benefits of peer support in professional development, teams should remain together and active for as long as practical and possible. When necessary, teams that have lost members should be combined to form new teams, while keeping students originally on the same team together, to maintain active team learning, support, and reflection until graduation from their program(s).

### Program Assessment

We believe evaluation of the program should be multifaceted. First, reflections should be graded based on writing/communication skills (50%) in addition to the extent of critical reflection exhibited (50%). In our opinion, *critical* reflection is exhibited when one recognizes how their thoughts and behaviors do not match their humanistic and professional values; experiences perplexity, doubt, hesitation, or mental difficulties; and begins to decide how better to align their values, thoughts, and behavior (25, 30). In contrast, reflection is limited when one thinks about an issue, critiques thoughts, and behavior (one’s own or others) and even suggests clichés on how best to think or behave; but no *critical* reflection is exhibited. Using these definitions, we found good correlation between grades assigned by different faculty members to the same written reflections (*r* = 0.92) (31). In our view, grades on these written reflections should constitute a portion of the grades earned in all courses and rotations during the academic term in which the reflections are performed.

Such efforts are feasible and need not unduly increase the workloads of students or faculty members (Table 1). For example, each student in our medical (about 200 students) and pharmacy (about 300 students) biochemistry courses performed at least 4 h of service learning per term, and they met with their student teams every 3 weeks to discuss each person’s service and reflections on it. Team meetings and student and team critical reflections extended over a total of seven months. All of the resultant student and team reflections were graded by the faculty after each of these team meetings, and the grades constituted a portion of the students’ grades for the courses. More than 93% of medical student teams (25) and 80% of interdisciplinary teams of pharmacy and prospective medical or dental students (28) performed critical thinking and reflection numerous times in at least one set of these individual and team written reflections.

**Table 1:** Effect size (ES) (*r*-values) for improvements of patient/relationship-centered characteristics in undergraduate medical students after continuous support by their teams of classmates for the indicated time periods.

Duration and location of indicated intervention	ES (*r*-values)	*p*-Values
**Five years in Belgium (13)**		
Scores of six measures considered together	0.91	0.004

**Third year in the US (15)**		
Reflection scores	0.445	<0.0001
Empathy scores	0.16	0.035 (One-tail *t*-test)

**Seven months in the US (25)^a^**		
Patient-centered scores^b^	0.61	0.004
Empathy scores (increase owing to reflection on community service)	0.44	0.04
Empathy scores (compared with prior class that did not perform community service)	0.80	<0.001

*^a^In this study, teams submitted their individual and team written reflections on service learning (or other challenges of medical education) every 3 weeks, and these reflections were graded by faculty members. This work was not detrimental to students’ academic performances and, in fact, increased these performances in our course, as is well documented in the literature for education in general (see text)*.

*^b^Patient/practitioner orientation scale*.

In our opinion, the program also should be assessed using validated surveys of patient/relationship-centered attitudes. For example, to evaluate our courses we have used the Jefferson Scale of Empathy (factors include “perspective-taking” and “compassionate care”), the Groningen Reflection Ability Scale (factors include “self-reflection,” “empathetic reflection,” and “reflective communication”), and the Jefferson Scale of Attitudes Toward Interprofessional Collaboration (factors include “working relationship” and “accountability”) (3, 18, 32). Other surveys are also available: no fewer than 64 valid instruments exist to assess teamwork among undergraduate health-care professional students (33). In the biochemistry courses discussed earlier, both student empathy scores and their attitudes toward interprofessional collaboration improved dramatically in association with student and team critical reflections on course-based community service (25, 28).

## Conclusion

The ability of clinicians to empathize with their patients has a tangible effect on patient well-being and wellness, leading to marked improvement in quantitative outcomes as well as qualitative measures of satisfaction and comfort. However, empathy scores deteriorate in most health-care professional students especially during the difficult first year of clinical training. Slowing or—better yet—eliminating this decline can be accomplished through long-term and continuous evidence-based interventions to support health-care professional students, especially when most vulnerable to the hidden curriculum in their programs. These long-term efforts to foster self-awareness and inward observation have been effective at not merely lessening empathy loss but actually advancing empathic behaviors and attitudes. In our opinion, heightened empathy and professional behavior among undergraduate health-care students promote public health, especially in association with service learning.

## Author Contributions

LV wrote the first draft including outlines for some sections. BS and NM wrote at least one of those outlined sections.

## Conflict of Interest Statement

The authors declare that the research was conducted in the absence of any commercial or financial relationships that could be construed as a potential conflict of interest.
